# Israeli Acute Paralysis Virus Infection Leads to an Enhanced RNA Interference Response and Not Its Suppression in the Bumblebee *Bombus terrestris*

**DOI:** 10.3390/v8120334

**Published:** 2016-12-19

**Authors:** Kaat Cappelle, Guy Smagghe, Maarten Dhaenens, Ivan Meeus

**Affiliations:** 1 Department of Crop Protection, Faculty of Bioscience Engineering, Ghent University, Coupure Links 653, 9000 Ghent, Belgium; guy.smagghe@ugent.be; 2 Department of Pharmaceutics, Faculty of Pharmaceutical Sciences, Ghent University, Ottergemsesteenweg 460, 9000 Ghent, Belgium; maarten.dhaenens@ugent.be

**Keywords:** Israeli acute paralysis virus, cricket paralysis virus, RNA interference (RNAi), viral suppressors of RNAi, *Bombus terrestris*, bee health

## Abstract

RNA interference (RNAi) is the primary antiviral defense system in insects and its importance for pollinator health is indisputable. In this work, we examined the effect of Israeli acute paralysis virus (IAPV) infection on the RNAi process in the bumblebee, *Bombus terrestris*, and whether the presence of possible functional viral suppressors could alter the potency of the host’s immune response. For this, a two-fold approach was used. Through a functional RNAi assay, we observed an enhancement of the RNAi system after IAPV infection instead of its suppression, despite only minimal upregulation of the genes involved in RNAi. Besides, the presence of the proposed suppressor 1A and the predicted OrfX protein in IAPV could not be confirmed using high definition mass spectrometry. In parallel, when bumblebees were infected with cricket paralysis virus (CrPV), known to encode a suppressor of RNAi, no increase in RNAi efficiency was seen. For both viruses, pre-infection with the one virus lead to a decreased replication of the other virus, indicating a major effect of competition. These results are compelling in the context of *Dicistroviridae* in multi-virus/multi-host networks as the effect of a viral infection on the RNAi machinery may influence subsequent virus infections.

## 1. Introduction

No domain of life is exempt from the threat of viruses [1], which hijack the cellular metabolic pathways to produce the genomic material and proteins needed for their own replication [2]. In order to counter these attacks, organisms have developed various defense mechanisms. Whereas in mammals the antiviral response is predominately mediated through the interferon response [3,4,5], in plants, nematodes and arthropods, this defense is carried out mainly by the RNA interference (RNAi) pathway [6,7]. This pathway is triggered by double-stranded RNA (dsRNA), the replicative intermediate form of RNA viruses [8,9], which is recognized and cleaved by the protein Dicer-2 (Dcr-2) to produce small interfering RNAs (siRNAs). These siRNAs are loaded onto Argonaute-2 (Ago-2) within the RNA-induced silencing complex (RISC) which guides the siRNA to complementary viral RNA strands and cleaves them, thereby preventing the production of the associated viral proteins [10]. If the RNAi mechanism acts systemically, a viral sequence-specific signal can be spread to uninfected tissues, resulting in a, at least partially, protected status of these cells [11].

During evolution, viruses have not stood by idly while their hosts developed this RNAi defense mechanism. Viruses known to encode viral suppressors of RNAi (VSRs) have been found in plants [12] and insects [13,14,15,16,17,18]. These VSRs are often small proteins which exercise their function through various mechanisms such as dsRNA sequestering and Dcr-2 or Ago-2 binding. Within the *Dicistroviridae*, a family of positive single-stranded RNA viruses infecting arthropods, a 166 amino acids (AA) long protein, called 1A, has been proven to be a functional VSR in both the drosophila C virus [14] and the cricket paralysis virus (CrPV) [15]. Recently, the presence of a similar 1A protein has been suggested in another member of the *Dicistroviridae*, Israeli acute paralysis virus (IAPV). The proof of its functionality was based on reduced virus titers after silencing the 1A region, compared to targeting the non-coding 5’ internal ribosome entry site (IRES) region [19], indicating the need of 1A for virulence. This method might be insufficient by itself, as 1A is a post-translation product and confounding effects like dsRNA target accessibility were not considered. Another small open reading frame (ORF), tentatively named *orfX* (or *pog*), has been predicted in IAPV and its close relatives but not in CrPV [20,21], and the presence of the resulting protein has been confirmed for IAPV in honeybees [22] but not for *Solenopsis invicta* virus 1 (SINV-1) [23]. As of yet, no functionality has been attributed to this putative 94 AA protein. VSRs can be very potent in the inhibition of the RNAi mechanism and therefore important immunosuppressive virulence factors. The actual virulence of a virus, defined as the relative capacity to cause damage in a host, depends on the balance between the power of the suppressor and the capacity of the RNAi mechanism of the host to limit the replication of the virus and, hence, the production of this inhibitor.

The aim of this study was to investigate to what extent IAPV can influence RNAi efficiency, and to which direction this balance will sway, in the bumblebee *Bombus terrestris*. This virus is known to pose an important health danger to pollinators such as honeybees and bumblebees [24,25] and is a target for RNAi-based antiviral therapeutics [26]. As an extra control, CrPV, with its known VSR in *Drosophila*, was included. CrPV has not been reported as a problematic infection of the pollinator community and has a broad host range as it was reported to infect species within the insect orders of Heteroptera, Diptera, Lepidoptera and Hymenoptera, at least in experimental conditions [27,28,29]. Within the concept of virus multi-host dynamics [30], the presence of VSRs can severely impact the virus virulence in different hosts, as the immunosuppressive capacity is dependent on the host immune strategies and their sensitivity towards VSRs. Also within the same host species, VSRs can influence the virulence of co-infecting viruses [31], an important feature now that multi-virus reports in pollinators are emerging [32,33].

In order to evaluate the effect of IAPV infection on RNAi efficiency in *B. terrestris*, we used a dual approach. First, a proteomic analysis was performed to confirm the translation of the VSR 1A and predicted OrfX proteins using the data-independent acquisition method with high definition mass spectrometry (HDMS^E^). Second, the RNAi efficiency after IAPV and CrPV infection was determined. An assay was developed in which bumblebees were infected with a fixed amount of viral particles and after an incubation period injected with dsRNA targeting *peptidylprolyl isomerase A* (*ppia*), a gene known to remain stable during virus infection [34]. Silencing levels were evaluated using reverse transcription quantitative PCR (RT-qPCR), along with expression levels of the RNAi core genes, *dcr-2* and *ago-2*, and the systemic RNAi genes, *ninaC*, *egghead* and *sid-1* [11].

## 2. Materials and Methods

### 2.1. Bumblebee Rearing and Injections

All experiments were performed using 5-to-10 day old workers, age fixed within each experiment, collected from *Bombus terrestris* colonies (Biobest NV, Westerlo, Belgium). Several workers were collected from the colonies and verified to be virus-free by PCR [35]. One or two workers were collected from each colony and randomly distributed over the microcolonies for the experiments. These microcolonies were placed in an incubator at 30 °C, 60% relative humidity and in continuous darkness and fed with sugar water (50% *w*/*v*, BIOGLUC^®^; Biobest NV) and gamma-irradiated pollen (Soc. Coop. Apihurdes, Pinofranqueado-Cáceres, Spain). Prior to injections with virus- or dsRNA-containing solutions, the bumblebees were anaesthetized by placing them on ice in a plastic container for 5 min. The bumblebees were then injected through the abdominal intersegment membrane between the second and third segment using an Femtojet Microinjector (Eppendorf, Hamburg, Germany). A volume of 5 µL was used for all virus solutions (and the appropriate controls), while dsRNA was injected in a volume of 20 µL.

### 2.2. Virus Production

IAPV was produced by injecting 160 virus-free bumblebees with 500 IAPV particles and waiting three days for the virus to amplify within the body. Then the bumblebees were crushed in 10 mM phosphate buffer (pH 7.0) supplemented with 0.02% diethyldithiocarbamate. The resulting suspension was centrifuged for 15′ at 800× *g* and 4 h at 100,000× *g* (4 °C). The resulting pellet was resuspended in 6 mL demineralized water. Subsequent dilutions were in phosphate buffered saline (PBS). Contamination of other common bumblebee viruses such as acute bee paralysis virus (ABPV), Kashmir bee virus (KBV), slow bee paralysis virus (SBPV), chronic bee paralysis virus (CBPV), deformed wing virus (DWV), Varroa destructor virus-1, Sacbrood virus and black queen cell virus, was determined to be less than 0.1% of the IAPV level by RT-qPCR [36] and the stock was negative for CrPV (determined by PCR).

CrPV was produced within the Schneider-2 (S2) cell line, which was tested to be negative for flock house virus, *Drosophila* melanogaster X virus, *Drosophila* melanogaster American nodavirus, *Drosophila* melanogaster totivirus and *Drosophila* melanogaster birnavirus [37]. 40 µL of CrPV inoculum (10^6^ particles/µL) was used to infect 40.10^6^ S2 cells and 15 h later the resulting viral particles were obtained by repeated freezing and thawing in a ultrafreezer (−70 °C) and subsequently applied to 300.10^6^ cells. 15 h later the resulting viral suspension was cleared of cell debris by centrifuging twice: 15′ at 800× *g*, followed by 30′ at 20,000× *g*. The viral suspension was cleaned further by ultracentrifuging 2 h, at 4 °C, at 100,000× *g* in a 15% sucrose gradient. The pellet was collected, resuspended in PBS and tested negative for IAPV, ABPV, KBV, DWV and SBPV using PCR [35].

For both viruses, the concentration of viral particles was estimated by transmission electron microscopy (CODA-CERVA, Brussels, Belgium). Briefly, Alcian blue-treated grids were deposited on a 15 μL drop of solution for 10 min and rinsed two times with water. Afterwards, the grids were stained for 10 s on a drop of 2% uranyl acetate (Agar Scientific, Stansted, UK), blotted and air-dried. The samples were imaged in bright field (BF) mode using a Tecnai Spirit TEM (FEI, Hillsboro, OR, USA) with Biotwin lens configuration operating at 120 kV. Five micrographs were recorded per sample using a 4K x 4K CCD camera (Eagle, FEI) at a magnification of 30,000 times.

### 2.3. dsRNA Synthesis

The dsRNA was prepared using the MEGAscript RNAi Kit (Life Technologies, Carlsbad, USA) according to the manufacturer’s specifications. The T7-DNA products used as dsRNA template were generated during a PCR reaction (five cycles using an annealing temperature of 55 °C followed by 25 cycles at 60 °C) using cDNA of bumblebee workers and the appropriate T7-primers (Table 1). For preparation of dsGFP, a linearized plasmid containing the green fluorescent protein (GFP) sequence was used as template. The dsRNA was eluted in 50 µL hot nuclease-free water and after concentration measurement, stored at −20 °C.

### 2.4. RNA Extraction, cDNA Synthesis and RT-qPCR

RNA was isolated using the RNeasy Mini Kit (Qiagen, Hilden, Germany) according to the manufacturer’s instructions and treated with the Turbo DNA-free kit (Life Technologies). For cDNA synthesis, 500 ng RNA was used in each reaction, performed with the SuperScript II Reverse Transcriptase Kit (Life Technologies). The RT-qPCR reactions were performed in duplicate in a C1000 Touch Thermal Cycler (Bio-Rad, Hercules, CA, USA) using the GoTaq qPCR Master Mix (Promega, Madison, WI, USA). Each reaction was composed of 1 μL of each primer (10 μM), 10 μL of GoTaq Master Mix and 8 μL of cDNA (diluted 1:40 or 1:100). The amplification conditions were 95 °C for 5 min followed by 40 cycles of 95 °C for 30 s, and 60 °C for 60 s. As reference genes *60S ribosomal protein L23* (*rpl23*) and *polyubiquitin B* (*ubi*) were used for normalization of the data [34]. Amplification efficiency and specificity of each primer set were evaluated using standard and melting curves. The results were analyzed using qbase^+^ software (Biogazelle, Zwijnaarde, Belgium). Reference gene stability was evaluated using the geNorm M value and the coefficient of variation on the normalized relative quantities (CV) values generated by the software. The thresholds for the M and CV values were set at 0.5 and 0.2 respectively for within-tissue comparisons and 1 and 0.5 for between-tissues comparisons [38]. An overview of the primers used in this work is given in Table 1. All primers were published [34] or designed using primer3 [39].

### 2.5. RNA Iinterference (RNAi) Efficiency Experiments, RNAi Gene Expression Levels and Pre-Infection Experiments

In order to test the effect of viral (IAPV or CrPV) presence on the RNAi efficiency, an assay was developed, containing three treatments. As a reporter gene, the endogenous *ppia* was chosen as it remains stable in the presence of IAPV [34]. Bumblebees belonging to the control group, with the purpose of determining the baseline *ppia* level, were injected with 5 µL PBS and 24 h later with 20 µg dsGFP (baseline control group) [36,40]. A second group was injected with 5 µL PBS and 20 µg dsPPIA and used to assess the RNAi efficiency in absence of the virus (RNAi control group). The virus treatment consisted of a first injection of 500 IAPV or 10^6^ CrPV particles, followed by an injection of 20 µg dsPPIA (virus treatment group). Because of a slower replication of CrPV the time between virus infection and dsRNA treatment was extended to 48 h and not longer to limit the effect of a bumblebee age difference. This way, there is an equal time between the end of the experiment and the onset of death for both viruses. Each group consisted of 7–9 individuals (*n*). Within one group, all bumblebees were placed in the same microcolony. This institutes a potential problem of pseudoreplication, however, the stable climatic conditions and ad libitum feeding limit the risk of microcolony effects. Moreover, the chosen reporter gene *ppia* is very stable (tested over different ages, infection levels, and tissues), thus, any large effects are very unlikely to be the result of pseudoreplication. Brain, fat body, midgut and ovaries were dissected 48 h after dsRNA treatment and stored separately in 350 µL RLT buffer. In order to assess the expression levels of the RNAi genes 24 h after IAPV treatment, a separate experiment was set up (*n* = 8–10). For the pre-infection experiments, similar timelines were used as in the RNAi efficiency assays. In a first experiment, injection with 500 particles of IAPV was followed by injection of 10^6^ particles of CrPV 24 h later (*n* = 9–10), in the other 10^6^ particles of CrPV were injected and 48 h later, 500 particles of IAPV (*n* = 11–15). Viral titers were analyzed using RT-qPCR 48 h after the second virus infection.

### 2.6. Statistical Analysis

All statistical analyses of RT-qPCR data were performed within SPSS (Version 23; IBM, Armonk, NY, USA). For experiments that evaluated viral titers, non-parametric methods were used (Friedman/Wilcoxon signed rank tests for dependent samples, Mann-Whitney U tests for independent samples). In the functional RNAi assay and the evaluation of the RNAi gene levels, the data was log_2_ transformed as advised in [38] after which the data satisfied the normality and homoscedasticity assumptions for analysis of variance (ANOVA) and Student’s *t*-test testing.

### 2.7. High Definition Mass Spectrometry (HDMS^E^)

Fifteen bumblebees were injected with 500 IAPV particles and the ovaries were dissected after three days at standard rearing conditions. However, the workers were not given pollen as to limit the development of the ovaries. The ovaries were pooled per three individuals and crushed in liquid nitrogen. Two third of the resulting powder was stored at −80 °C for mass spectrometry, while one third was dissolved in RLT buffer (RNeasy Mini Kit; Qiagen) awaiting RNA extraction (for confirmation of infection). The powdered sample was resuspended in an Eppendorf protein LoBind tube in 45 µL 0.5 M triethylammonium bicarbonate (TEABC; Sigma-Aldrich), supplemented with sodium dodecyl sulfate (SDS, 0.1% *v*/*v*) and acetonitrile (ACN; 10% *v*/*v*), in the presence of Halt Protease and Phosphatase Inhibitor Cocktail (Perbio Science, Erembodegem, Belgium) and 100 U of benzonase nuclease (Sigma-Aldrich, St. Louis, MO, USA). The sample was sonicated on ice and centrifuged. The protein concentration was determined using the Bradford assay and 2.5 µg of protein was reduced in 0.5 M TEABC by adding 1 µL reducing agent and incubating for 30 min at 60 °C, followed by alkylation using a 10 mM methyl methanethiosulfonate for 10 min at room temperature (RT). The samples were digested in 1 mM CaCl_2_ by adding trypsin/lysC (25:1 protein/enzyme ratio; Promega). They were placed overnight at 37 °C and evaporated, after which the sample was resuspended in 0.1% formic acid (FA). 100 ng of peptide sample, spiked with 25 fmol Hi3 standard and 25 fmol MSPD standard, was injected of each sample.

Peptides were analyzed using a nanoscale UPLC system (nanoAcquityUPLC, Waters Corporation, Milford, CT, USA) coupled to a Synapt G2-Si mass spectrometer (Waters Corporation). Peptides were first trapped on a 180 µm × 20 mm C18 Trap column in 0.1% FA and separation was performed on a HSS C18 1.8 m, 100 m × 250 mm analytical column at a flow rate of 300 nL/min and a temperature of 45 °C. A 0.1% formic acid with 4% dimethyl sulfoxide (DMSO) in water was used as mobile phase A and 80% ACN containing 0.1% formic acid as mobile phase B. Peptides were separated for 60 min at 1%–40% solvent B, for 1 min 40%–85% solvent B. After 7 min of rinsing using 85% solvent B the column was re-equilibrated to the initial conditions. Eluted peptides were analyzed in positive mode ESI-MS using HDMS^E^ with a collision energy look up table as described in [41] and the data were post acquisition lock mass corrected with [Glu1]-Fibrinopeptide B. The mass spectrometer was operating in HDMS^E^ which couples high-efficiency ion mobility separations (IMS) with time of flight (TOF) mass analysis. The spectral acquisition time of a low and elevated energy scans was 0.6 s over an *m*/*z* range of 50–2000.

Data analysis of the raw files obtained from the Synapt G2-Si was performed in Progenesis QI (Nonlinear Dynamics; Version 2.0). The PLGS Threshold Inspector was used to iterate through a range of apex low energy and high energy settings to determine the optimal threshold for protein identification. Peptides with charges from two to five were retained following the data normalization in the Progenesis. For protein identification, the LC-MS data were processed and searched by Protein Lynx Global SERVER (PLGS, Version 3.0.2; Waters Corporation) with peptide tolerance and fragment tolerance set to auto. Protein identifications were obtained by searching a compiled database of UniProtKB/Swiss-Prot entries belonging to IAPV supplemented with the cRAP database (laboratory proteins and dust/contact proteins) and sequences of spiked standard proteins, which was concatenated to a randomized decoy database. For peptide identification, the following search criteria were selected: (i) trypsin as digestion enzyme; (ii) up to one missed cleavage allowed; (iii) fixed methylthio-cysteine and variable methionine oxidation, deamidation at asparagine and glutamine. The false discovery rate (FDR) for protein identification in PLGS was set to 1% threshold. Only proteins identified with at least two unique peptides were further considered.

## 3. Results

### 3.1. Israeli Acute Paralysis Virus (IAPV) Genome Structure and HDMS^E^

**Figure 1:**
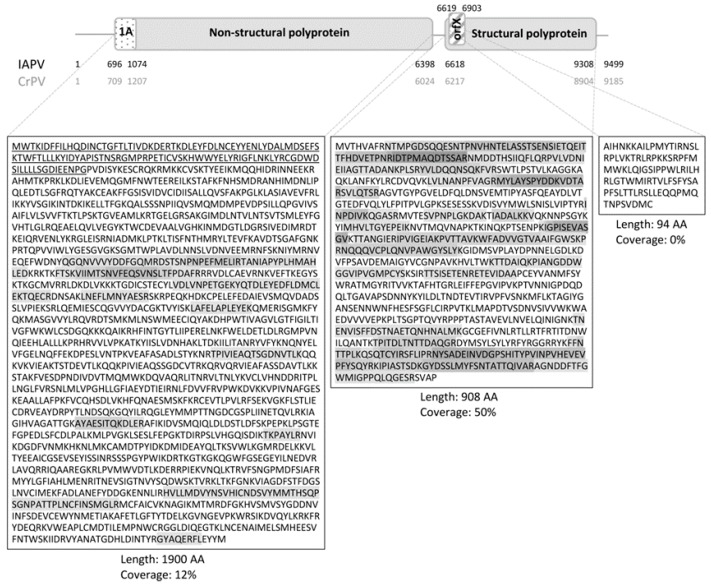
Israeli acute paralysis virus (IAPV) genome organization and high definition mass spectrometry (HDMS^E^) coverage. The *Dicistroviridae* genome consists of two open reading frames (ORFs), coding for a non-structural and a structural polyprotein. The first stretch of amino acids (AA) in the former polyprotein, upstream from Stop-Go translational cleavage site NPG^▼^P, form the 1A protein (dotted). In IAPV the length of this protein is 126 AA, whereas in cricket paralysis virus (CrPV) it is 166 AA. In the +1 frame of the 5′ end of the second ORF of IAPV, the possible *orfX* (striped) was predicted (not present in CrPV). Start and stop nucleotide positions of the genome, the ORFs and the 1A and OrfX coding sequences are given in black for IAPV and grey for CrPV. (Poly)protein sequences are given and the location of the 1A protein is underlined. HDMS^E^ coverage is indicated by the darkness of the AA letter code’s background. Polyprotein length and HDMS^E^ coverage are given underneath the protein sequences.

An overview of the genome structure of the *Dicistroviridae* IAPV and CrPV is given in Figure 1 and the HDMS^E^ coverage is denoted by the darkness of the polyprotein sequences’ background. The coverage differed between the (poly)proteins; the 64 detected peptides covered 50% of the AA sequence of the structural polyprotein (48 peptides), whereas only 12% of the non-structural protein was detected (16 peptides). Within the OrfX protein, none of the seven predicted peptides (at least 6 AA long) after trypsin digestion were detected. The presence of 1A would be confirmed by detecting the Stop-Go translational cleavage at the NPG^▼^P site, but the resulting non-tryptic CGDWDSILLLLSGDIEENPG peptide was not observed.

### 3.2. Virus Distribution in Bumblebee Tissues

**Figure 2:**
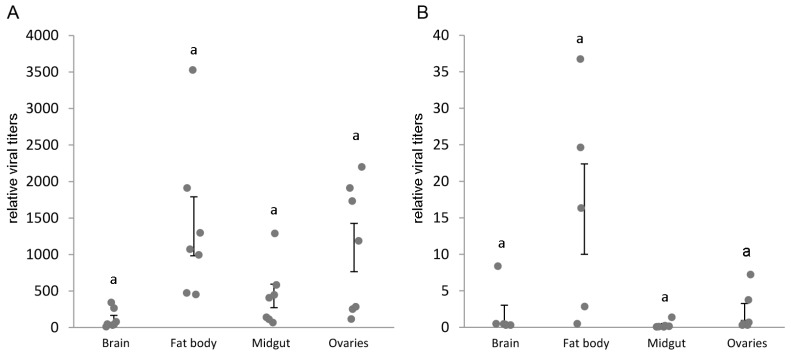
IAPV and CrPV tissue distribution in different bumblebee tissues. Seven bumblebees were injected with 500 particles of IAPV (**A**) or 10^6^ particles of CrPV (**B**) and the viral titers, after 72 h or 96 h, respectively, were evaluated using RT-qPCR. The dots represent the individual viral titers, relative to the normalized level of the reference genes *rpl23* and *ubi.* The error bars show the SEM on the mean. Statistical analysis was performed using the non-parametric Friedman rank test as samples from the same individuals are not independent, resulting in significantly different viral titers over the tissues (IAPV: Q-stat. = 13.29, *p* = 0.001; CrPV: Q-stat. = 10.92, *p* = 0.003). Comparisons between treatments were made using Wilcoxon signed rank tests, none of which differed significantly on an α = 0.05 level after Bonferroni correction. In general, there was a considerably large variation within the tissues, but the higher viral titers were found in the fat body and ovaries for IAPV, and fat body for CrPV.

Infections with IAPV and CrPV showed a similar relative distribution over the tissues, with higher viral titers in the fat body and for IAPV also in the ovaria, and lower viral titers in brain and midgut (Figure 2). However, there was a remarkably large variation within the tissues. Interesting to note is that the average viral titer of IAPV in the fat body was around 1400 times the normalized level of the reference genes *rpl23* and *ubi*, whereas for CrPV it was only 16 times, even with an infection dose of only 500 particles of IAPV and 10^6^ of CrPV. CrPV replication was confirmed by showing a 11,000-fold increase in viral titers 4 days after infection compared to the input viral titer minutes after injection (*n* = 5 at each time point, data not shown).

### 3.3. RNAi Efficiency in Bumblebee Tissues

**Figure 3:**
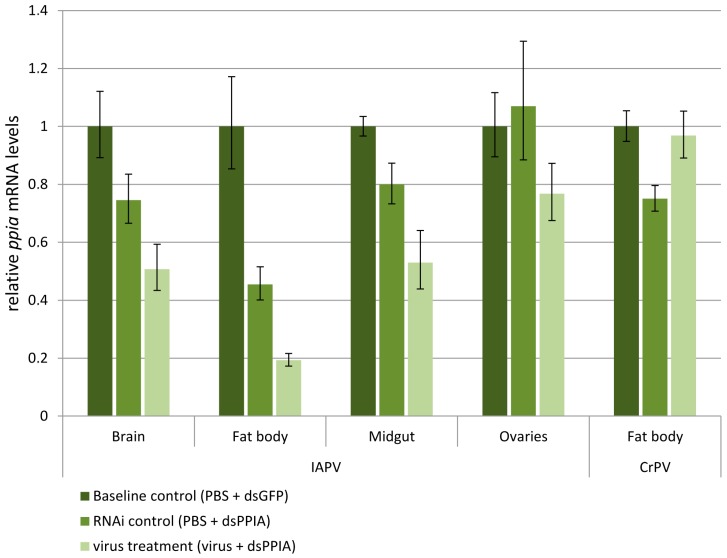
RNA interference (RNAi) efficiency in different bumblebee tissues, with and without IAPV or CrPV infection. Seven to nine bumblebees were treated with 20 µg dsPPIA (or dsGFP in the baseline control). In the virus treatment 500 particles of IAPV or 10^6^ particles of CrPV were administered 24 or 48 h, respectively, beforehand. The effect of the treatment on the silencing of the reporter gene *ppia* was evaluated using RT-qPCR 48 h after dsRNA application. All data was normalized to the *ppia* levels in the baseline control. Statistical analysis was performed using analysis of variance (ANOVA) on log_2_ transformed data with Tukey’s HSD for post-hoc comparisons between the treatments. The columns represent the treatment mean ± SEM (on a linear scale) and statistical differences on an α = 0.05 level are denoted by different letters above the column. Comparing the RNAi control (middle green) to the baseline control (dark green) represents the RNAi efficiency in the different tissues in the absence of viruses, with a significant RNAi event only happening in the fat body. IAPV virus treatment (light green) showed an increased silencing efficiency in brain, fat body and midgut, whereas CrPV treatment resulted in a diminished RNAi efficiency as the expression levels rise to a comparable level as in the baseline control.

In the *ppia*-targeting assays, the RNAi efficiency could be evaluated in both the absence and presence of the viruses. The expression levels of *ppia* in the RNAi control compared to the baseline control measure RNAi efficiency in a virus-free condition, whereas the levels in the virus treatment give an indication of how the virus influences the RNAi efficiency (Figure 3). In the ‘IAPV’ experiment, ANOVAs over the three treatments showed significant differences in *ppia* levels in the brain (*F*_2,22_ = 7.417, *p* = 0.003), fat body (*F*_2,20_ = 29.317, *p* < 0.001) and midgut (*F*_2,22_ = 11.218, *p* < 0.001), but not in the ovaries (*F*_2,22_ = 0.788, ns). In the following sections, the ovaries will not be discussed further. The other tissues will collectively be referred to as ‘responsive tissues’. For the ‘CrPV’ experiment, the *ppia* levels in the fat body were also statistically different (*F*_2,25_ = 5.864, *p* = 0.008).

Different silencing efficiencies were observed between the selected bumblebee tissues after 48 h in the absence of viruses (comparison between baseline control and RNAi control in Figure 3). For both the IAPV and the CrPV experiment, the expression levels of *ppia* dropped significantly in the fat body (IAPV: *p* = 0.002 (Tukey’s HSD); CrPV: *p* = 0.011 (Tukey’s HSD)). However, the *ppia* levels dropped by 55% in the ‘IAPV’ experiment, whereas they only declined by 25% in the CrPV experiment (note that there can be no effect of virus presence in this comparison). In the brain and midgut, the *ppia* levels were slightly lowered, but there was too much biological variation in the IAPV experiment to confirm an RNAi event (*F*_2,22_ = 7.417, *p* > 0.05 (Tukey’s HSD) and *F*_2,22_ = 11.218, *p* > 0.05 (Tukey’s HSD) respectively).

### 3.4. Virus Infection Alters RNAi Efficiency

When IAPV was administered 24 h before the dsRNA treatment (virus treatment in Figure 3), a significant silencing effect on the reporter gene *ppia* was noticed in all three tissues of brain (*p* = 0.002 (Tukey’s HSD)), fat body (*p* < 0.001 (Tukey’s HSD)) and midgut (*p* < 0.001 (Tukey’s HSD)) compared to the baseline control, and in the case of the fat body and the midgut also a significant silencing compared to the RNAi control (*p* = 0.001 (Tukey’s HSD) and *p* = 0.013 (Tukey’s HSD), respectively) The *ppia* levels in brain, fat body and midgut were lowered to 51%, 19% and 53% of their original levels, respectively. These results indicate an enhancement of the RNAi effect after IAPV infection in all tissues except for the unresponsive ovaries.

In the case of CrPV, only the fat body was analyzed as it was the only tissue showing a significant silencing effect without IAPV presence, which is necessary for confirming RNAi inhibition. The virus was administered to the bumblebees 48 h before dsRNA treatment (instead of 24 h for IAPV). This later time-point was chosen because injection of CrPV resulted in slower replication and later onset of mortality of the bees. We have determined the onset of death to occur at 4 days post infection (dpi) and 5 dpi for IAPV and CrPV, respectively (data not shown), so this alteration promotes similar virus–host interactions at the moment of dsRNA treatment and RT-qPCR evaluation. Forty-eight hours after dsRNA treatment this resulted in a significant increase in *ppia* levels compared to the RNAi control (*p* = 0.036 (Tukey’s HSD)). Moreover, no significant silencing could be observed compared to the baseline control (*p* > 0.05 (Tukey’s HSD)), indicating that the RNAi system might have become impaired after CrPV infection.

### 3.5. Expression Levels of Genes Involved in RNAi after Virus Infection

**Figure 4:**
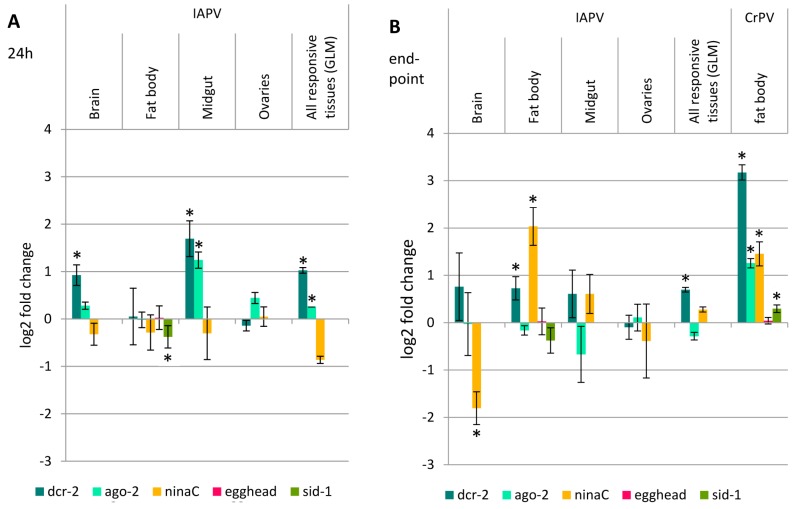
Fold change of selected RNAi genes upon IAPV or CrPV infection. The expression levels of the RNAi core genes *dcr-2* and *ago-2* and the systemic RNAi genes *ninaC*, *egghead* and *sid-1* were evaluated at the moment of dsRNA application (**A**) and at the endpoint of the RNAi efficiency experiment (**B**) using RT-qPCR. Statistical analysis was performed using the Student’s *t*-test on log_2_ transformed data. For the general linear model (GLM), only the responsive tissues, brain, fat body and midgut, were used. The columns represent the mean ± SEM on a log_2_ scale, normalized to the control, and statistical differences on an α = 0.05 level are denoted by an asterisk. In the case of IAPV, *dcr-2* showed a significant upregulation in some tissues at both 24 and 72 h, and also over all responsive tissues. However, its fold change is considerably smaller than the nine-fold change of *dcr-2* levels 96 h after CrPV infection. *ago-2* expression levels were augmented in the midgut 24 h after IAPV infection and over all responsive tissues, but no effect was seen at the assay endpoint. For IAPV, the systemic RNAi genes showed occasional alterations in expression levels, however, over all responsive tissues the changes were not significant, whereas for CrPV, *ninaC* and *sid-1* are significantly upregulated.

In order to assess whether the altered RNAi efficiency after virus infection is due to an upregulation of the genes involved in the RNAi pathway, the expression levels of *dcr-2, ago-2, ninaC, egghead* and *sid-1* were evaluated 24 h after IAPV infection (the moment the dsRNA is administered; virus injected compared to PBS injected; Figure 4A) and at the endpoint of the assay (72 h for IAPV and 96 h for CrPV; virus treatment compared to RNAi control; Figure 4B).

First, the RNAi core genes, *dcr-2* and *ago-2*, were assessed in all tissues 24 h post IAPV infection (Figure 4A). A significant upregulation of *dcr-2* was observed in the brain and midgut (*t*_9.334_ = −2.572, *p* = 0.029; *t*_8.497_ = −2.275, *p* = 0.05, respectively). A general linear model (GLM) analysis over the responsive tissues showed a significant effect on *dcr-2* over the different tissues (*F*_1_ = 5.638, *p* = 0.021). The expression of *ago-2* was increased in midgut (*t*_17_ = −4.432, *p* < 0.001) and in the GLM (*F*_1_ = 6.356, *p* = 0.015). At the endpoint of the assay (Figure 4B), a significant *dcr-2* upregulation was detected in the fat body (*t*_14_ = −2.314, *p* = 0.036) and a similar, but more variable and not significant upregulation in the brain and midgut (*t*_7.68_ = −0.996, *p* > 0.05 and *t*_6.90_ = −1.165, *p* > 0.05, respectively). The GLM over the responsive tissues showed a significant effect of *dcr-2* (*F*_1_ = 5.744, *p* = 0.024). The expression levels of *ago-2* remained unaltered in all tissues.

When looking at the systemic RNAi genes *ninaC*, *egghead* and *sid-1* in the fat body, only the former was upregulated (*t*_14_ = −4.23, *p* = 0.001). Therefore, the expression levels of *ninaC* were also determined in the other tissues. However, *ninaC* was significantly downregulated in the brain (*t*_12.10_ = 2.775, *p* = 0.016) and no alteration was observed in the midgut (*t*_14_ = −1.195, *p* > 0.05) nor in the GLM over all responsive tissues (*F*_1_ = 0.715, ns).

For CrPV, there was a much more evident upregulation of the RNAi core genes *dcr-2* and *ago-2* in the fat body after 96 h (*t*_16_ = −16.056, *p* < 0.001 and *t*_16_ = −10.706, *p* < 0.001, respectively), as well as the systemic RNAi genes *ninaC* and *sid-1* (*t*_16_ = −2.978, *p* = 0.009 and *t*_16_ = −2.931, *p* = 0.010, respectively). The *dcr-2* upregulation after CrPV infection is over nine-fold, whereas for IAPV over all responsive tissues not even two-fold. The expression levels of *egghead* remained unaltered (*t*_10.032_ = −0.195, *p* > 0.05) (Figure 4B).

### 3.6. IAPV/CrPV Levels after Pre-Infection

**Figure 5:**
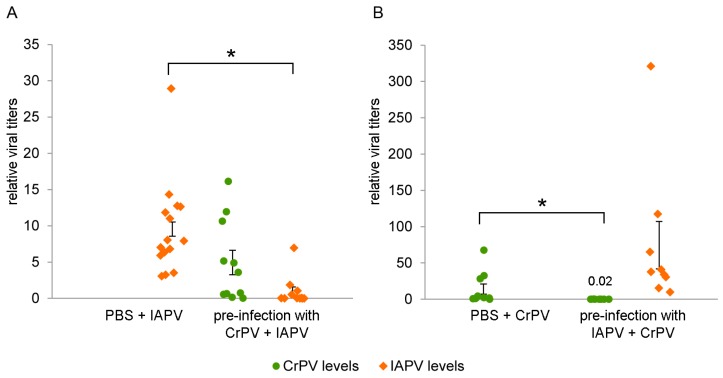
Virus levels of IAPV/CrPV after pre-infection with CrPV/IAPV. Bumblebees were either pre-infected with 10^6^ particles of CrPV and 48 h later with 500 particles of IAPV (**A**) or pre-infected with 500 particles of IAPV and 24 h later with 10^6^ particles of CrPV (**B**). The viral titers were analyzed 48 h after the second viral infection using RT-qPCR. The dots represent the individual data points relative to the normalized level of the reference genes *rpl23* and *ubi*, and the SEM of the mean is depicted by the error bars; *, relevant statistical differences on an α = 0.05 level (Mann-Whitney U test).

In both cases, there is a clear decrease in viral titers in the fat body compared to the control after pre-infection with the other virus. Pre-infection with CrPV for 48 h reduced the IAPV levels 90% (Mann-Whitney U = 6, *p* < 0.0001, Figure 5A), whereas pre-infection with IAPV for 24 h reduced the CrPV levels with almost 99% (Mann-Whitney U = 2, *p* = 0.0005, Figure 5B). Lower IAPV levels in Figure 5A are due to the evaluation occurring 48 h post-infection (pi), whereas in Figure 5B they are analyzed 72 h pi.

## 4. Discussion

Virus presence has been argued to be associated with variability of RNAi within a species as it can have a dual interaction with the RNAi defense system [42]. On one hand, the presence of viral dsRNA fragments activates the RNAi pathway, on the other hand, VSRs can inhibit this powerful antiviral defense system. The RNAi defense potency, in turn, influences viral replication and may determine the survival chances of the host. In this work, we examined whether the presence of an immunosuppressive virulence factor of IAPV can tip the scale towards one of these two opposites in the bumblebee.

A first confirmation of the presence of a VSR in IAPV could come on a proteomic level, from the detection of VSR-specific peptides. The ovaries were selected for HDMS^E^ analysis because (1) the viral titers in the ovaries were considerably high; (2) protein extraction from the fat body is challenging; and (3) the ovaries do not contain complex microbiota like the midgut does, which might confound the analysis. The HDMS^E^ on IAPV-infected ovaries resulted in a coverage that is comparable to a similar setup for IAPV in *Apis mellifera* [43] with a higher coverage for the structural polyprotein than the non-structural polyprotein. In order to confirm the presence of the 1A VSR, one specific peptide with the alternative Stop-Go translational cleavage [44] needed to be detected. This was not the case, but the lower presence of the corresponding polyprotein or peptide characteristics like the isoelectric point or peptide length could have impeded detection. The same reasons might also explain why no peptides belonging to the possible out-of-frame protein OrfX were found, but in this case, there are multiple peptides that theoretically should be detectable.

From the HDMS^E^ result, by itself, it is not possible to confirm with any certainty the absence of these putative VSR proteins in IAPV-infected cells. However, when coupled with the RNAi efficiency data, where the presence of VSR in IAPV should suppress RNAi efficiency, it can be concluded that, even if the 1A or OrfX proteins are produced in the cell during IAPV infection, and even if they are functional VSRs, their functionality is being outweighed by the RNAi system or that the RNAi system of bumblebees is insensitive towards the VSRs of IAPV.

Not only could the predicted VSR of IAPV not suppress the RNAi efficiency in *B. terrestris*, but also an enhancement of the RNAi activity was observed. The fact that a diminished RNAi effect is seen using the same setup for CrPV ensures the efficacy of the *ppia* assay. An argument could be made that the difference between the experiments could be explained by the longer incubation of CrPV. We believe it was appropriate to extend the viral pre-infection duration because of the lower virulence of CrPV while a 24-h age difference between the bumblebees will probably not affect their capacity for RNAi. The more important question is determining how this IAPV-induced enhancement of RNAi activity can be explained.

A first explanation could be that virus infection causes the *ppia* levels to drop because of a disturbed cellular machinery. However, we collected ample evidence that this is not the case (Figure S1). Another reason could be that the upregulation of the RNAi core genes that is sometimes seen after viral infection in insects [36,45,46] could make this pathway more potent. We noticed a slight (~2-fold) upregulation of *dcr-2* after IAPV infection in some tissues which also showed an increased RNAi efficiency. In contrast, for CrPV a notably larger upregulation (~9-fold) was seen. It seems that this upregulation of *dcr-2* does not determine the outcome of the RNAi pathway, possibly because the VSR 1A is acting on Ago-2, a component downstream of Dcr-2 [15]. Interestingly, we have previously shown that *dcr-2* silencing does not diminish IAPV replication in bumblebees, possibly because of the too robust replication of IAPV [36]. Second, host response to IAPV infection could enhance the systemic properties of the RNAi system, facilitating the spread of the silencing signal and resulting in an increased silencing efficiency. This argument seems less adequate as for IAPV, nothing really stood out (except some changes in *ninaC* expression but they are variable over the different tissues). Again, for CrPV, more significant positive fold changes are noticed (i.e., *ninaC* and *sid-1* in the fat body). The fact that an upregulation of *ninaC* was also seen in the fat body after IAPV treatment might indicate a role of the fat body in triggering a systemic RNAi response, but additional experiments are needed before any conclusions can be drawn on this. The exact mechanism behind the IAPV-induced RNAi efficiency remains inconclusive; possibly one or more genes that drive RNAi efficiency fell out of our selection or a yet unknown RNAi signal-spreading mechanism is activated by the presence of IAPV.

The viral influence on the RNAi machinery, a key immune response against viral infections, has great implications for host–virus dynamics. It is important to repeat that IAPV is a known pathogen of various pollinating hymenopterans [24,25], whereas CrPV has been detected once in honeybees [29] and appears to infect bumblebees, but seems to have a wide experimental host range outside of the pollinators [27,28]. Please note that CrPV identification in honeybees was based on serological tests available at the time and thus misidentification due to cross-reactivity with related viruses cannot be excluded. The tissue distribution pattern of the two viruses IAPV and CrPV is remarkably similar, both having the highest viral titers in fat body, and a considerably large variation between individuals. In mosquitoes, the fat body has been suggested to be the primary amplifying tissue for the positive ssRNA viruses *West Nile virus* [47] and *Dengue virus* [48], where it is thought to be the intermediate station between the primary infection in the midgut and the spreading towards the other tissues. Like others have noted before, this is remarkable as the fat body is also considered a particularly immunocapable tissue [49] and we have observed this to be a tissue exhibiting a significant RNAi response. Although IAPV induced the antiviral defense system, IAPV reached 100-fold higher viral titers starting from a 2000-fold lower injection dose. This could indicate that RNAi potency is not the primary determining factor in viral infectivity. Cell entry and manipulation of the host cell’s protein synthesis or other immune pathways could be more decisive for explaining viral replication dynamics, especially since IAPV has adapted to infect bees, so it could have evolved mechanisms for evading the immune response other than the suppression of RNAi.

Our findings are also interesting in light of RNAi-therapeutics development and the use of RNAi as a research tool in functional genomics. The RNAi efficiency in the absence of a virus varies between the different tissues examined, with a significant silencing effect only occurring in the fat body and ovaries which are unresponsive. It is conceivable that the virus could evade the therapeutic in tissues which are insensitive to RNAi, such as the ovaries which showed relatively high viral titers in this study. Variations in RNAi efficiency over insect tissues have also been found in *Anopheles gambiae* with salivary glands that are refractory to conventional RNAi [50] and in many lepidopterans for which RNAi experiments are generally more successful in the hemocoel-surrounding tissues and less in the epidermal tissues (overview in [51]). An insensitivity to RNAi in the ovaria, as observed here, was also seen in *Schistocerca gregaria* [52] in the honeybee *Apis mellifera* after siRNA injection [53], and was attributed to a lack of dsRNA uptake in this tissue in *Locusta migratoria* [54]. It needs to be noted that the dsRNA was delivered by injection and had direct access to most tissues through the hemolymph, therefore bypassing the midgut barrier. The results also affirm the need for an evaluation at the tissue level instead of at the whole-body level in similar experiments as both viral titers and RNAi efficiency differ considerably between tissues.

An interesting question emerging from these results is the functionality of the CrPV 1A in bumblebees, a hymenopteran species, which was previously shown to be active in the dipteran *Drosophila* [15]. Although there is a statistically significant reduction in RNAi efficiency, the fact that the RNAi effect in absence of the virus is not that large prevents confirming its functionality within the bumblebee. As a single bee host is often infected with multiple viruses, VSRs could have competitive or synergistic effects. Pre-infection with a VSR-coding virus, such as CrPV, could facilitate subsequent infection and colonization, while an infection of IAPV would make it more difficult for other viruses to co-infect as the RNAi system becomes more efficient. Carrillo-Tripp et al. showed that pre-treatment with CrPV-1A could induce cytopathogenic effects of Deformed wing virus, persistently present in the AmE-711 cell line [31]. Our results show a large reduction in viral titers after pre-infection with the other virus. In the case of IAPV pre-infection, this could be the result of a combination of an enhanced RNAi machinery and competition for the same host resources. IAPV, under normal circumstances, replicates extremely efficiently in bumblebees. For CrPV, however, this similar reduction of IAPV titers suggest that the VSR functionality, which was dubious in the RNAi assays, is not relevant. The mere presence of CrPV, and its saturation of the translational machinery, limits IAPV replication. It would be interesting to examine how this pre-infection with CrPV influences IAPV virulence in natural infections. In conclusion, we can state that there is a complex interaction between viruses and the RNAi defense mechanism of the insect host. Therefore, VSR functionality cannot be inferred from virus relatedness and needs to be taken into account when looking at virulence and multi-virus/multi-host dynamics.

## Figures and Tables

**Table 1:** Overview of the primers used in this work.

Primer Name	Sequence (5′–3′)	Length (bp)	Eff. (%)
IAPV_q F	CCATGCCTGGCGATTCAC	203	97–102
IAPV_q R	CTGAATAATACTGTGCGTATC		
CrPV_q F	AAACGCAAAAACAGCGAAAC	110	103–104
CrPV_q R	CACATCAAGCACCAAAGCAT		
rpl23_q F	GGGAAAACCTGAACTTAGGAAAA	143	86–99
rpl23_q R	ACCCTTTCATTTCTCCCTTGTTA		
ubi_q F	GGTATTTGGATGCCAGTGATTT	129	94–96
ubi_q R	ATGGGCATTTCTACCCCTTTTA		
ppia_q F	TCGTAATGGAGTTGAGGAGTGA	132	84–94
ppia_q R	CTTGGCACATGAAGTTTGGAAT		
dcr-2_q F	TGGTCAAAACATCAAGAACAACCA	211	93–97
dcr-2_q R	GATCGGGGCCATACGAACAT		
ago-2_q F	CCGAATGTGGACAATGCTTA	181	95–102
ago-2_q R	AACGGGCAAAGGTGTGATTA		
sid-1_q F	CGAGCCCATCAACGGTAGAA	160	94–107
sid-1_q R	CGAGCCAAATCACAAACGGA		
ninaC_q F	GCGAAACCATCTGGAGGATA	112	91–106
ninaC_q R	ACTCTGTTAGCCGCATCGTT		
egghead_q F	ACCGGAGGACTTAGTTGGAA	122	93–97
egghead_q R	TGCGGAAAGGAAAGAAATGT		
GFP_T7 F	TAATACGACTCACTATAGGGTACGGCGTGCAGTGCT	495	/
GFP_T7 R	TAATACGACTCACTATAGGGTGATCGCGCTTCTCG		
ppia_T7 F	TAATACGACTCACTATAGGGCACTGGTGGAAGGTCCATCT	388	/
ppia_T7 F	TAATACGACTCACTATAGGGAAGGGAAAATGGTGATGATTAGAA		

bp, base pairs; Eff., minimal and maximal amplification efficiencies over the different experiments; IAPV, Israeli acute paralysis virus (EU436443.1); CrPV, cricket paralysis virus (KP974707.1); *rpl23*, *60S ribosomal protein L23* (XM_003400707.2); *ubi*, *polyubiquitin B* (XM_003402262.2); *ppia*, *peptidylprolyl isomerase A* (XM_003402218.2); *dcr-2*, *dicer-2* (XM_012307737.1); *ago-2*, *argonaute-2* (XM_012312881.1); *sid-1*, *systemic RNA interference (RNAi) deficient 1-like* (XM_012315164.1); *ninaC*, *neither inactivation nor afterpotential C* (XM_003393094.2); *egghead*, *beta-1,4-mannosyltransferase egghead* (XM_012321382); GFP = *green fluorescent protein* (M62654.1).

## Supplementary Materials

The following figure is available online at www.mdpi.com/1999-4915/8/12/334/s1, Figure S1: Additional experiments illustrating the fact that *ppia* levels remain stable after virus infection or dsRNA treatment.
