# Supplementary material for: Israeli Acute Paralysis Virus Infection Leads to an Enhanced RNA Interference Response and Not Its Suppression in the Bumblebee Bombus terrestris
Source: Viruses. 2016 Dec 19;8(12):334. doi: 10.3390/v8120334 (PMC5192395; doi:10.3390/v8120334)
Supplement: Supplementary file 1 [file viruses-08-00334-s001.pdf]

Supplementary Materials: Israeli Acute Paralysis Virus Infection Leads to an Enhanced RNA Interference Response and Not Its Suppression in the Bumblebee *Bombus terrestris*

Kaat Cappelle, Guy Smagghe, Maarten Dhaenens and Ivan Meeus

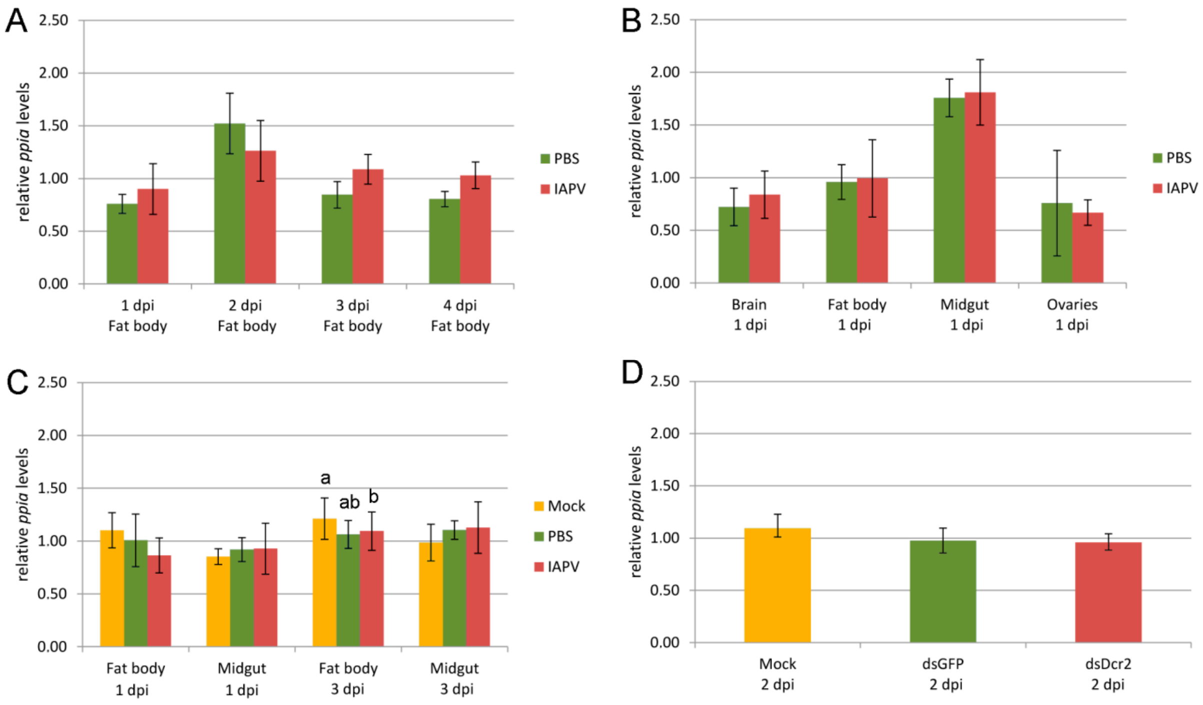

Supplementary Figure S1. Additional experiments illustrating the fact that *ppia* levels remain stable after virus infection or dsRNA treatment. All bumblebees were injected with 500 particles of IAPV (in 5 µL), 5 µL of PBS, 20 µg of dsRNA or not injected (i.e. mock treatment). The effect of the treatment on the expression of the reporter gene *ppia* was evaluated using RT-qPCR, normalized to *rpl23* levels. (**A** and **B**) Statistical analysis was performed using Student's t-test. The stability of *ppia* during the course of an IAPV infection is shown in (**A**), whereas (**B)** proves stability over the different tissues. (**C** and **D**) Statistical analysis was performed using Analysis of Variance (Tukey's HSD post-hoc comparisons; on $\log_2$ transformed data. The columns represent the treatment mean ± SD, and statistical differences on an $\alpha = 0.05$ level are denoted by different letters. (**C**) shows the reproducibility of these results, with a mock infection as extra control and (**D**) depicts the stability of this gene after non-targeting dsRNA treatment. In one instance, there is a minor downregulation of *ppia* 3 days after IAPV infection, but only compared to the mock treatment, so this is probably caused by stress after the injection together with biological variation. All other appropriate comparisons are not statistically different.

© 2016 by the authors, licensee MDPI, Basel, Swizerland. This article is an open access article distributed under the terms and conditions of the Creative Commons Attribution (CC-BY) license (http://creativecommons.org/licenses/by/4.0/).
